# Supplementary material for: Neuroprotective and Regenerative Effects of Growth Hormone (GH) in the Embryonic Chicken Cerebral Pallium Exposed to Hypoxic–Ischemic (HI) Injury
Source: Int J Mol Sci. 2022 Aug 13;23(16):9054. doi: 10.3390/ijms23169054 (PMC9409292; doi:10.3390/ijms23169054)
Supplement: Supplementary file 1 [file ijms-23-09054-s001.zip › ijms-1839795-supplementary.pdf]

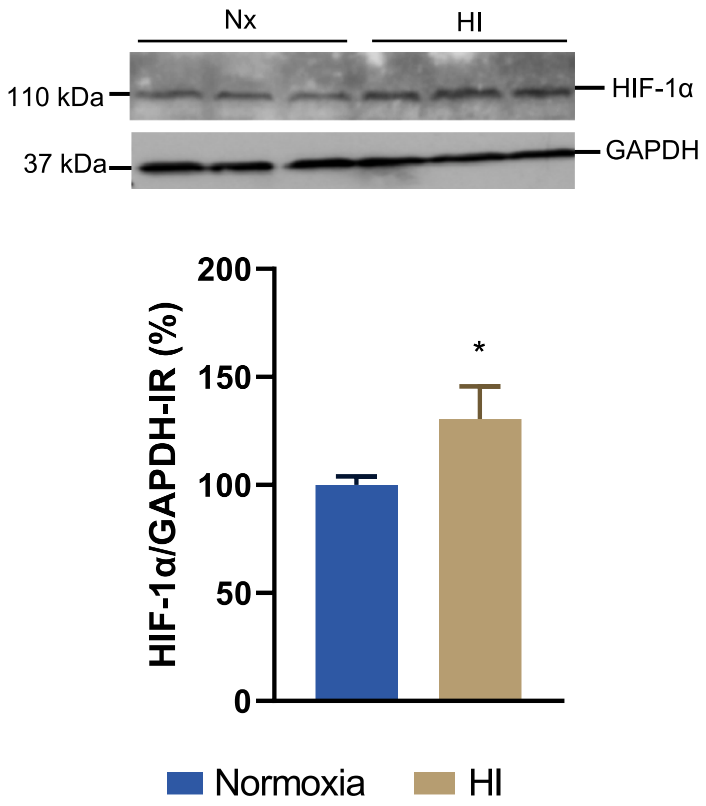

Figure S1. HIF-1α immunoreactivity (IR) in pallial tissue of chick embryos exposed to hypoxia-ischemia (HI) injury. Densitometric analysis of immunoblot stained for HIF-1α immunoreactivity (IR). The values were corrected and normalized with GAPDH-IR. Bars represent mean ± SEM (n = 6 chick embryos per group). The asterisk (*) indicate significant differences between groups ($p < 0.05$) as assessed by an unpaired Student's t test.
